# Climate change effects on desert ecosystems: A case study on the keystone species of the Namib Desert *Welwitschia mirabilis*

**DOI:** 10.1371/journal.pone.0259767

**Published:** 2021-11-08

**Authors:** Pierluigi Bombi, Daniele Salvi, Titus Shuuya, Leonardo Vignoli, Theo Wassenaar

**Affiliations:** 1 Institute of Research on Terrestrial Ecosystems, National Research Council, Monterotondo, Rome, Italy; 2 Department of Health, Life and Environmental Sciences, University of L’Aquila, Coppito, L’Aquila, Italy; 3 Gobabeb Namib Research Institute, Walvis Bay, Namibia; 4 Department of Science, University of Roma Tre, Rome, Italy; 5 Department of Agriculture and Natural Resources Sciences, Namibia University of Science and Technology, Windhoek, Namibia; Estacion Experimental de Zonas Aridas, SPAIN

## Abstract

Deserts have been predicted to be one of the most responsive ecosystems to global climate change. In this study, we examine the spatial and demographic response of a keystone endemic plant of the Namib Desert (*Welwitschia mirabilis*), for which displacement and reduction of suitable climate has been foreseen under future conditions. The main aim is to assess the association between ongoing climate change and geographical patterns of welwitschia health, reproductive status, and size. We collected data on welwitschia distribution, health condition, reproductive status, and plant size in northern Namibia. We used ecological niche models to predict the expected geographic shift of suitability under climate change scenarios. For each variable, we compared our field measurements with the expected ongoing change in climate suitability. Finally, we tested the presence of simple geographical gradients in the observed patterns. The historically realized thermal niche of welwitschia will be almost completely unavailable in the next 30 years in northern Namibia. Expected reductions of climatic suitability in our study sites were strongly associated with indicators of negative population conditions, namely lower plant health, reduced recruitment and increased adult mortality. Population condition does not follow simple latitudinal or altitudinal gradients. The observed pattern of population traits is consistent with climate change trends and projections. This makes welwitschia a suitable bioindicator (i.e. a ‘sentinel’) for climate change effect in the Namib Desert ecosystems. Our spatially explicit approach, combining suitability modeling with geographic combinations of population conditions measured in the field, could be extensively adopted to identify sentinel species, and detect population responses to climate change in other regions and ecosystems.

## Introduction

Climate change is one of the strongest threats for ecosystems worldwide. Variations in the density of species, range changes, and extinction events have been documented at local and global level [1–3]. Furthermore, changes in species diversity, ecosystem functioning, and service provision are expected for the future as a consequence of climatic pressures on natural populations [4–6]. In Africa, deep impacts by climate change have been forecasted for animals [7–9], plants [10–12], and biodiversity in general [4,13].

Desert ecosystems are predicted to be one of the most vulnerable ecosystems to global climate change [14–16]. Rising temperature, decreasing rainfall, and increasing atmospheric CO_2_, are expected to strongly affect the structure and function of desert ecosystems [15,17]. Desert-adapted species are vulnerable to climate change [18] and among them endemic plant species are particularly susceptible to the loss of suitable habitat [19]. The negative effect of climate change on desert plants has been demonstrated worldwide [20].

**Fig 1:**
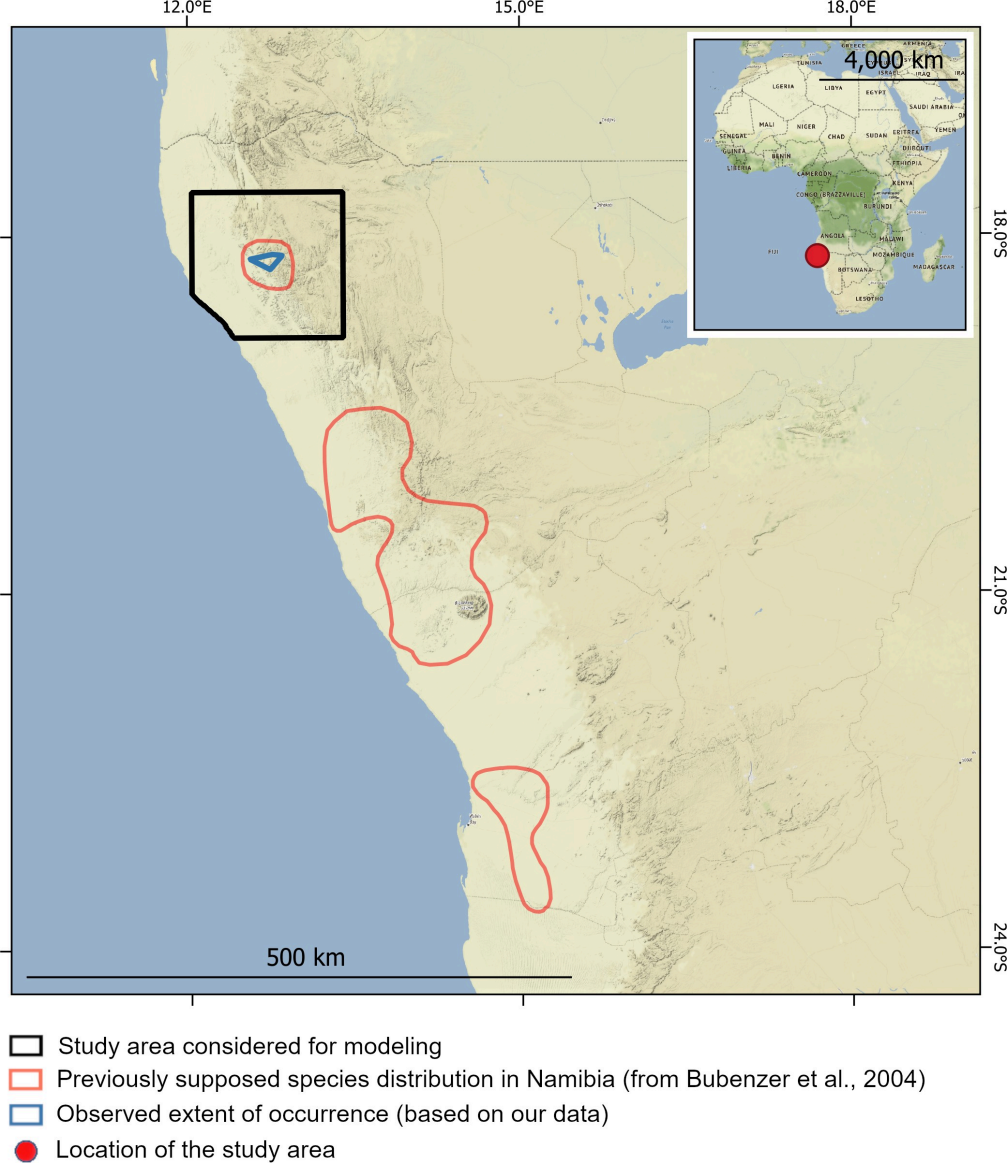
Distribution of *Welwitschia mirabilis* in Namibia and location of the study area. In the main map, the black polygon indicates the study area, the red polygons show the known species distribution, and the blue polygons represent the boundaries of the observed extent of occurrence in Northern Namibia. In the inset map the red circle indicates the general location of the study area in Africa. Map tiles by Stamen Design, under CC BY 3.0.

In the arid regions of southern Africa, projections of climate change impacts on species persistence indicate a high vulnerability of endemic plant diversity to climate change [19,21]. For example, climate-linked increases of mortality have been observed for the quiver tree (*Aloidendron dichotomum* Klopper & Gideon 2013; [22], and a potential decrease of climatic suitability was recently pointed out by Bombi [23] for welwitschia (common name for *Welwitschia mirabilis* Hooker 1863). *Welwitschia mirabilis* is regarded as a living fossil, representing an ancient lineage of gymnosperm plants, and it is recognized as a symbol of the Namib Desert biodiversity. This species has a peculiar morphology, being a long-living dwarf tree with only two leaves growing throughout its entire life [24]. This is also a keystone species for the Namib desert ecosystems, where it provides food, water, and refuge for many animal species, including mammals, reptiles, and insects [25,26]. *Welwitschia mirabilis* is endemic to the central and northern Namib Desert, ranging between the Kuiseb River in Namibia and the Nicolau River, north of Namibe, in Angola [27,28]. In this area, welwitschia plants occur in four separated sub-ranges, three in western Namibia (Fig 1) [29] and one in south-western Angola. Bombi [23] showed that populations living in the three Namibian subranges have experienced and will in the future face rather different climatic conditions. The same study predicted a significant reduction of climatic suitability in the northernmost Namibian subrange (which lies in a transition zone between desert and arid savanna) under current climate change scenarios. In particular, the ongoing rise of temperature can drive the local climate out of the realized niche for the northern populations, thus increasing their extinction risk [23]. Although these findings were potentially important for conservation planning, the study was based on low spatial resolution data (2.5 arcmin) available at the national scale, thus limiting its utility for targeting individual populations.

Many species respond to climate change by changing their distribution range [30–32]. These changes have been generally described as poleward and/or upward movements to track suitable temperature conditions along latitudinal and altitudinal gradients [33–35]. However, in many cases documented geographic patterns of response are complex and do not align with simple latitudinal and altitudinal shifts [36]. Indeed, the assumption of simple, uni-directional distribution shifts does not account for intricate interactions among temperature, precipitation, and species-specific tolerances and can lead to substantial underestimation of the effect of climate change on species distributions [37]. To overcome these drawbacks, one promising approach to quantify possible range changes is based on the comparison of the species-specific spatial pattern of climatic suitability variation (the expected responses), generated by predictive models, with the pattern of appropriate metrics of population conditions measured in the field (the observed responses) [38]. This approach can increase our ability to identify the effect of climate change on species dynamics.

The main aim of this study was to determine whether the observed geographic combination of population condition (plant health, reproductive status, and size) of welwitschia in northwestern Namibia is associated with ongoing climate change. Secondly, we tested if the same pattern follows a latitudinal or altitudinal gradient in agreement with the assumption of a poleward or upward range shift. More specifically, we wanted to first validate the projections of potential impacts of climate change on *W*. *mirabilis* with field-based data. Then, we wanted to assess whether the simple assumption of a poleward/upward range shift is suitable for detecting climate change effects. To do this, we compared the geographic combination of population conditions, measured in the field, with the expected pattern of response, estimated by ecological niche models. If climate change is affecting welwitschia populations, we expected the worst population condition in sites where climatic suitability is decreasing and the best where suitability is increasing. Moreover, if a poleward/upward range shift is the major response to climate change, we could expect a latitudinal or altitudinal trend in the observed patterns of response. Since potential divergent responses to climate change by intraspecific lineages have been observed before [39] and different realized niches were described for each distinct Namibian subrange [23], we focused on populations in the northern subrange and considered them as an independent ecological unit, with its own climatic niche and with its (sub)specific expected response. By testing our main and secondary hypotheses, we hope to inform the long-term conservation of *W*. *mirabilis* and further contribute to the scientific debate on the climate change impacts on biodiversity.

## Materials and methods

### Field data collection

During May 2019, we carried out a field expedition in the northernmost Namibian subrange of *W*. *mirabilis*, as defined by the ’Digital Atlas of Namibia’ [29], in order to obtain information relevant for the species conservation. This study was authorized by the National Commission on Research, Science and Technology of Namibia (Research Permit RCIV00032018) and performed in public lands, managed by the Orupembe, Sanitatas, and Okondjombo Communal Conservancies. During the expedition, we spent 10 full days, in a team of six persons, searching for welwitschia plants across the northernmost Namibian subrange by (1) driving at low speed along the available tracks (more than 330 km) while recording the presence of plants in a ~30 m wide transect on each side of the vehicle, and (2) walking across potentially suitable habitats (more than 65 km) in ~60 m wide transects on each side, in both valley bottoms and hill slopes. Doing this, we explored comprehensively more than 65 km^2^ (330 km x 30 m x 2 sides + 65 km x 60 m x 2 sides x 6 persons). The starting points and spatial extent of our walking transects were informed by the knowledge of our local team members, who have an intimate knowledge of the area. We are confident that the combination of local knowledge and systematic transects extending beyond the known range have allowed us to establish the extent and characteristics of the majority of this sub-range.

We collected detailed data on four categories of plant traits: plant location, health condition, reproductive status, and plant size. More specifically, we recorded the precise coordinates (using a handheld GPS) (1), the sex (2), and the presence/absence of cones (3) for almost all the individual plants we observed (just a few, unreachable plants were excluded). The health condition (4), the stem diameters (minimum (5) and maximum (6) along the two main axes of the stem), and the mean leaf length (along the curved trajectory of the leaves) (7) were measured in sites with a sufficient number of plants (> 25 for variable 4 and > 35 for variables 5–7) for reducing the effect of chance on categorical and numerical variables. In sites with more than 60 plants, we considered a random subset of ~60 plants. We ranked health condition on a four-point scale (dead, poor, average, good) based on leaf color (see S1 Fig). Although this is a relatively coarse scale, the brightness of the green color and the ratio of red/brown to green together are a remarkably consistent and accurate indicator of good health condition as measured by photosynthesis efficiency [40]. The green color of the leaf is associated with the chlorophyll content and the photosynthetic efficiency of the tissues [41,42], which is influenced by environmental stress [43,44]. An estimate of health condition such as the above is both a direct reflection of the environmental (including climatic) stress that the plant experiences and an index of the likelihood that its resistance to parasites might be compromised [45,46]. We expected that changes in local climate will be visible in its leaf color as a quick proxy of plant health.

### Observed pattern of response

For each welwitschia stand (defined *a posteriori*, through a GIS-based analysis, as a group of plants separated from the other groups by a distance larger than the intra-group mean distance), we calculated three categories of synthetic indicators of population response (derived from plant health, reproductive status, and size) from the field-measured data. For each stand, we calculated the proportion of plants that were dead or in poor, average, and good condition to the total number of plants in the stand. We also calculated the reproductive status (the proportion of plants in the stand that had cones) and the plant size (average stem major axis, stem minor axis, and leaf length). The presence/absence of cones were used as a proxy of population recruitment potential instead of other, more common methods (e.g. plant size) in order to gather a trend of the last few years (after 2000) in plants with an extraordinary low growth rate.

In order to test a previously proposed gradient in plant size, health condition, and reproductive status from hill slopes and valley bottoms [27], we compared these variables for plants growing in the drainage systems with those in steeper locations.

### Expected pattern of response

We used a spatially explicit approach based on ecological niche modeling to estimate the geographic combination of plant response expected as a consequence of climate change. To do this, we defined our study area as a bounding box three times larger than the latitudinal and longitudinal extent of the previously known subrange of welwitschia in northern Namibia [29]. Inside this study area, we fitted models on 1000 pseudo-presence/absence points by using historical (1950–2000) climate data from the WorldClim databank, version 1.3 [47] at the spatial resolution of 30 arcsec (about 1 km) in the *R*-based [48] *biomod2* Package [49]. In order to control the model-associated uncertainty, we adopted an ensemble forecasting approach [50]. In particular, we used Generalized Linear Models [51], Generalized Additive Models [52], Generalized Boosting Models [53], Classification Tree Analyses [54], Artificial Neural Network [55], and Random Forest [56] methods, which are widely used and recognized as robust methods.

Pseudo-presence/absence points were randomly generated across the study area and classified as presence or absence points based on their position inside or outside the species extent of occurrence, generated as a minimum convex polygon from our detailed distribution data. Doing this, we overcame the problems related to the small number of real sites of presence and to the spatial autocorrelation due to the non-homogeneous distribution of the real sites [57]. Multicollinearity among predictors was reduced by discarding those with variance inflation factor higher than five [58]. Thus, four variables were retained for modeling (i.e. Temperature Seasonality, Mean Temperature of the Driest 3-months period, Precipitation Seasonality, Precipitation of the Warmest 3-months period) (See S2 Fig). Each independent model was projected into the study area under historical climatic conditions and three-fold cross-validated by calculating the true skill statistic (TSS) [59]. Finally, we generated a single consensus model of historical suitability. More specifically, we used the six individual models to predict a continuous value of suitability (between 0 and 1), so the final consensus suitability is the TSS-weighted average of six values for each 30 arcsec pixel. Suitability ranges between 0 (no suitability) and 1 (perfect suitability). Future climate suitability was predicted by projecting the models into future climatic conditions across the study area. We used Global Climate Models (GCMs) elaborated by 19 research centers within the Coordinated Modelling Intercomparison Project Phase 5 (CMIP5), which represented the basis for the Fifth Assessment Report of the Intergovernmental Panel on Climate Change (IPCC-5thAR) [60]. GCMs were elaborated for the four Representative Concentration Pathways (RCP2.6, RCP4.5, RCP6.0, and RCP8.5) for 2050 (average for 2041–2060) and downscaled at the same spatial resolution as the historical climate data using WorldClim as baseline climate [61].All the 59 available scenarios of future (2050) climate from CMIP5 were utilized for projecting the models. We compared the historical climate data and the future climatic scenarios by calculating the Multivariate Environmental Similarity Surfaces (MESS) [62] in order to estimate the reliability of predictions. The MESS calculation estimates the extent to which the predictor variables in the study area are similar to the conditions experienced by the species in the presence sites. Negative MESS values indicate areas where at least one variable is outside the range of the experienced conditions and thus where model predictions can be less robust [62].

Suitability variation over time was calculated as the difference between future and historical suitability (Suitability variation = future suitability–historical suitability) and assigned to the observed plant stands on the basis of their location. Suitability variation ranges between -1 (perfectly historically suitable and perfectly unsuitable in the future) and 1 (perfectly historically unsuitable and perfectly suitable in the future). Following our approach, the predicted suitability variation represents an ongoing process, ranging between 2000 and 2050, and the population data collected in the field represent a snapshot approximately in the middle of this process.

### Association between observed and expected patterns

#### Test of model-based pattern

For each variable, we tested the linkage between the observed and the expected patterns of responses at the stand level (i.e. measured synthetic indicators *vs* modelled suitability variation) by adopting a null-model approach [63–65]. First, we quantified the observed correlation between measured values and expected suitability variation in the same sites (*r*_*obs*_) by calculating the Pearson r. Second, we generated in *R* (as all the other analyses) 30,000 random permutations of the measured values and calculated the simulated correlation with the expected suitability variation for each permutation (*r*_*sim*_). Third, we calculated the probability of the null hypothesis that the observed correlation was drawn at random from the distribution of the simulated correlations [66]. Finally, in order to control the familywise error rate due to multiple comparisons, we corrected our *p* values adopting the approach proposed by Benjamini & Hochberg [67]. These corrected *p* values (*p*_*corr*_) measure the level to which the suitability variation (corresponding to the expected response) due to climate change explains the actual responses observed in the different stands.

In addition, we tested whether the observed responses follow a general and simple geographic combination. We tested the hypothesis of a latitudinal (equator-to-pole) or altitudinal (low-to-high elevation) range shift, as if often assumed for detecting climate change impacts on species distributions. To do this, we adopted the same approach used for testing the linkage between the observed and the model-based expected patterns of responses. We contrasted each measured variable with the stand latitudes and altitudes. We quantified the correlation between the measured variable and the latitude/altitude, calculated the probability that the observed correlation comes randomly from the simulated correlations after 30,000 random permutations, and corrected our *p* values with the Benjamini & Hochberg [67] approach. With this approach, we assumed that populations at higher latitude/elevation should be in better general conditions (i.e., lower proportion of plants in poor conditions and of dead plants and higher proportion of plants in good conditions and of plants with cones) if climate change effect is following a simple geographic gradient. As a result, we obtained an estimation of the extent to which climate change effects can be explained as a simple geographic gradient.

## Results

Overall, we recorded 1330 plants within the known distribution of *W*. *mirabilis* in northern Namibia. These plants are clustered in 12 distinct stands, which are scattered across the central part of the known range at elevations between 806 and 991 m above sea level. On the basis of our field effort and the expert knowledge of our local team members, we are confident that the recorded/observed individuals, and their resulting extent of occurrence, represent the majority of plants in this northern-Namibian sub-range. The area of each recorded stand varied from 2000–825,000 m^2^ (for a total surface of about 1.5 km^2^) and the number of plants per stand varied between four and about 400. The extent of occurrence (estimated as the minimum convex hull) of welwitschia in the area covers about 215 km^2^ and the inter-stand distance varied from 1.8 to 30 km. Even though we searched extensively throughout the study area, we defined a markedly smaller extent of occurrence than the distribution map previously published for the species in northern Namibia [29], which was based on very general information.

**Fig 2:**
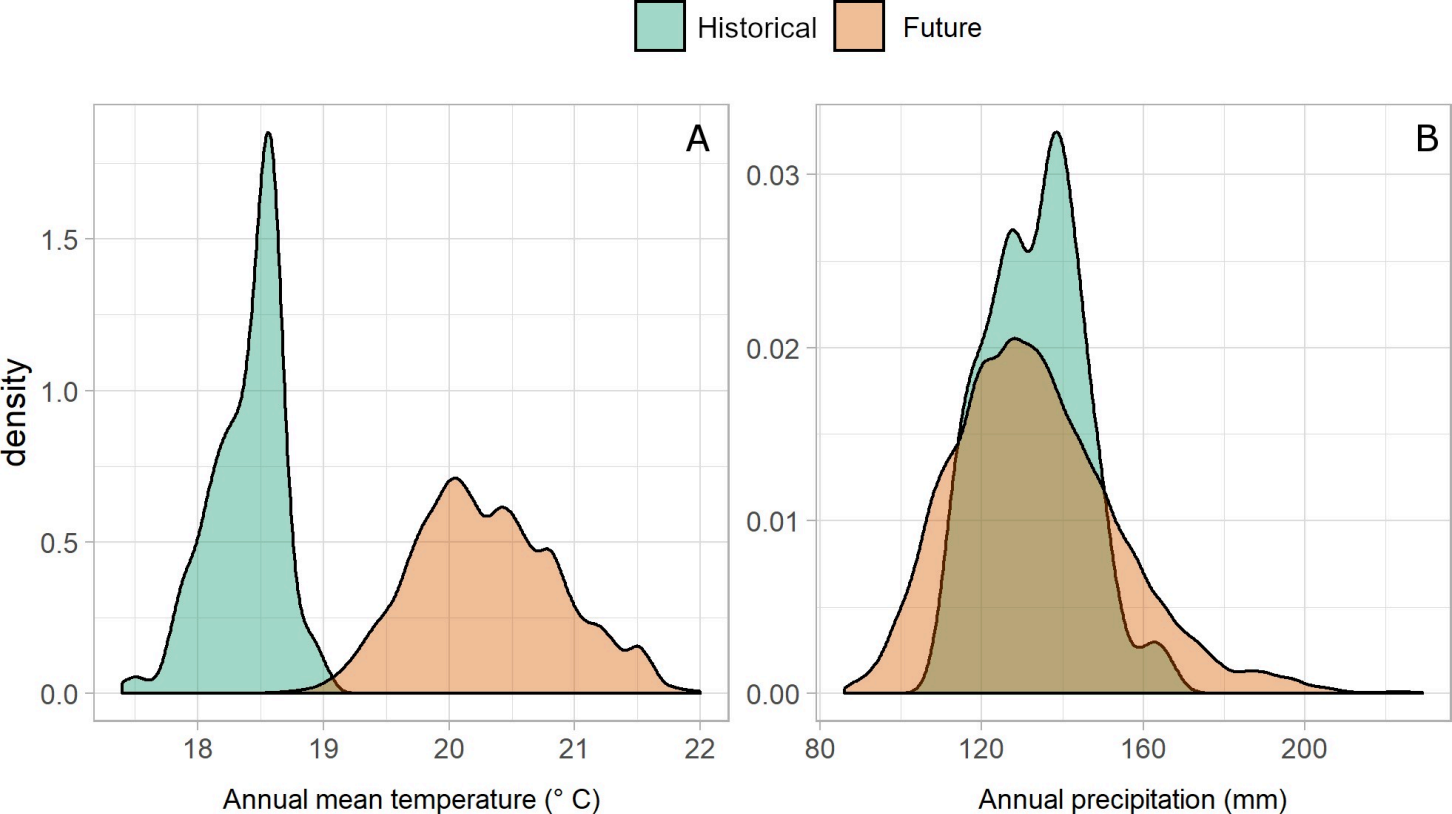
Historical and future climatic conditions in the welwitschia extent of occurrence. Density plot of historical data (in green) and expected future values (in orange) for annual mean temperature (A) and annual precipitation (B) in the welwitschia extent of occurrence. Density plots use kernel density estimates to show the probability density function of the variables.

The available climatic models revealed that the temperature historically available in the current extent of occurrence of welwitschia in northern Namibia is becoming almost completely unavailable in the same area (Fig 2A). In particular, annual mean temperature within the stands is rising about 1.5–2.5°C, with strong variations among the different CMIP5 scenarios. In contrast, the total annual precipitation is likely remaining relatively stable (Fig 2B), with small reductions or increases forecasted by different scenarios.

### Observed variation of plant parameters

**Fig 3:**
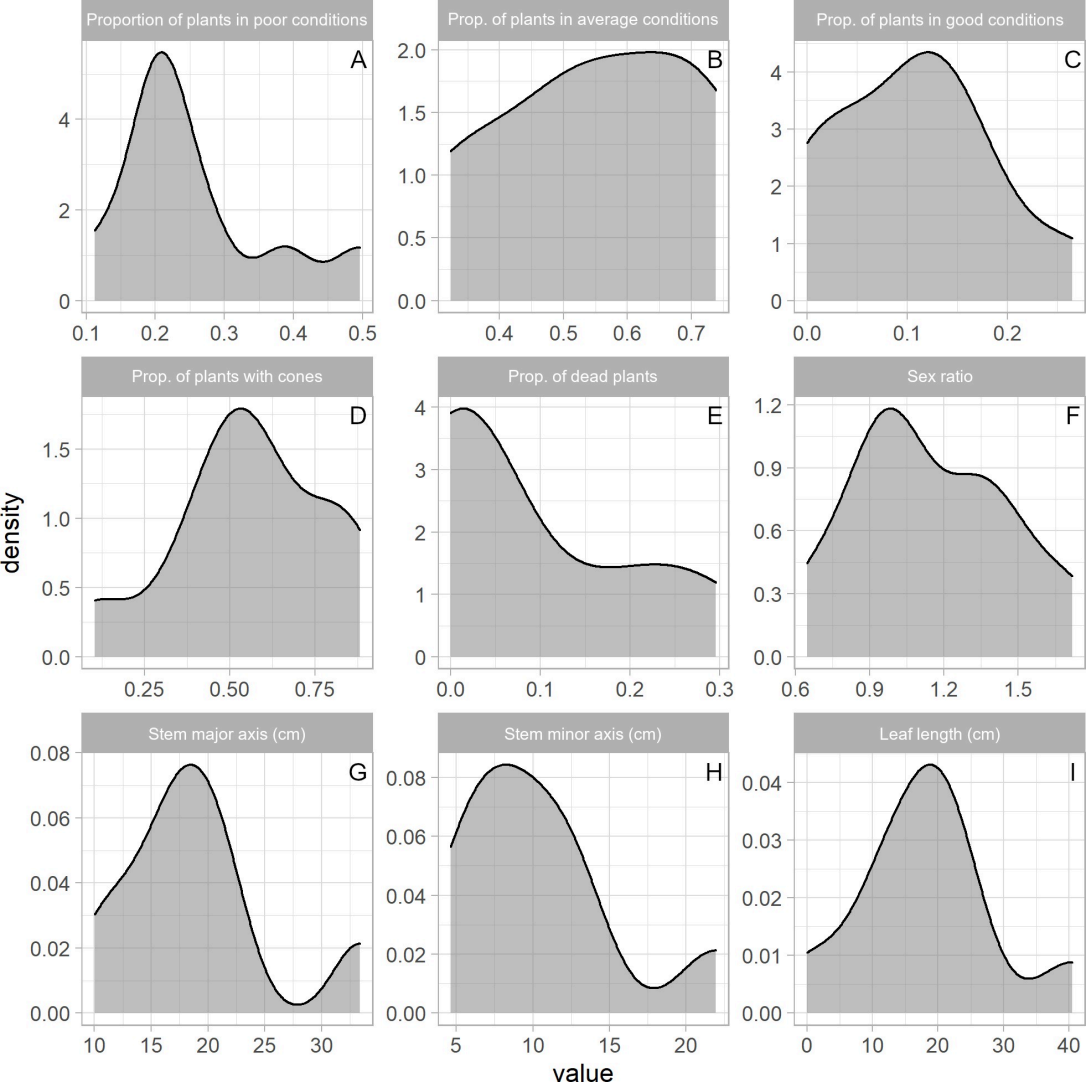
Observed variability of plant traits. Density plots of measured values for: proportion of plants in poor conditions (A), proportion of plants in average conditions (B), proportion of plants in good conditions (C), proportion of plants with cones (D), proportion of dead plants (E), sex ratio (F), stem major axis (G), stem minor axis (H), and leaf length (I). Density plots use kernel density estimates to show the probability density function of the variables.

The most common class of health condition was ‘average’, with 50% of all the plants and a range between 32% and 74% across individual stands being found in this status (Fig 3B). Plants in ‘poor’ condition were 32% (range: 11–50%), but only 10% of all plants were in a ‘good’ condition (range: 0–30%) (Fig 3A and 3C respectively). Seven percent of all plants were dead (range: 0–30%) and 56% (range: 10–90%) had cones (Fig 3E and 3D respectively). Not all individuals could be sexed, but among those that were, 56% were males, with a sex ratio (males/females) ranging between 0.6–1.7 across stands (Fig 3F). Stem major axis and stem minor axis were highly variable, ranging from 2 to 100 cm (18.8 ± 14.1 cm; range: 10–33 cm) and from 0.3 and 55 cm (10.3 ± 9.8 cm; range 4.6–22 cm), respectively (Fig 3G and 3H respectively). Leaf length varied from almost 0 cm (completely browsed plants) up to 93 cm (18.7 ± 13.4; range 11–40 cm) (Fig 3I) (see S1 Table).

The previously proposed difference between plants growing in valley bottoms and on hill slope (Kers, 1967) was not verified. Indeed, most of the tested variables were not significantly different for the two groups of plants (stem minor axis: Mann-Whitney *U* = 10773, *p* = 0.49; leaf length: *U* = 10364, *p* = 0.1968; health condition: Mann-Whitney *U* = 189, *p* = 0.32; reproductive status: *χ*^*2*^ = 0.05, *p* = 0.82). The plant stem major axis only was different (*U* = 13120, *p* = 0.02) but in the opposite direction respect to the proposed pattern, with plants on slopes bigger than plants on valleys (mean: 14 ± 7.85 cm and 11 ± 8.48 cm respectively).

### Expected pattern of species response

**Fig 4:**
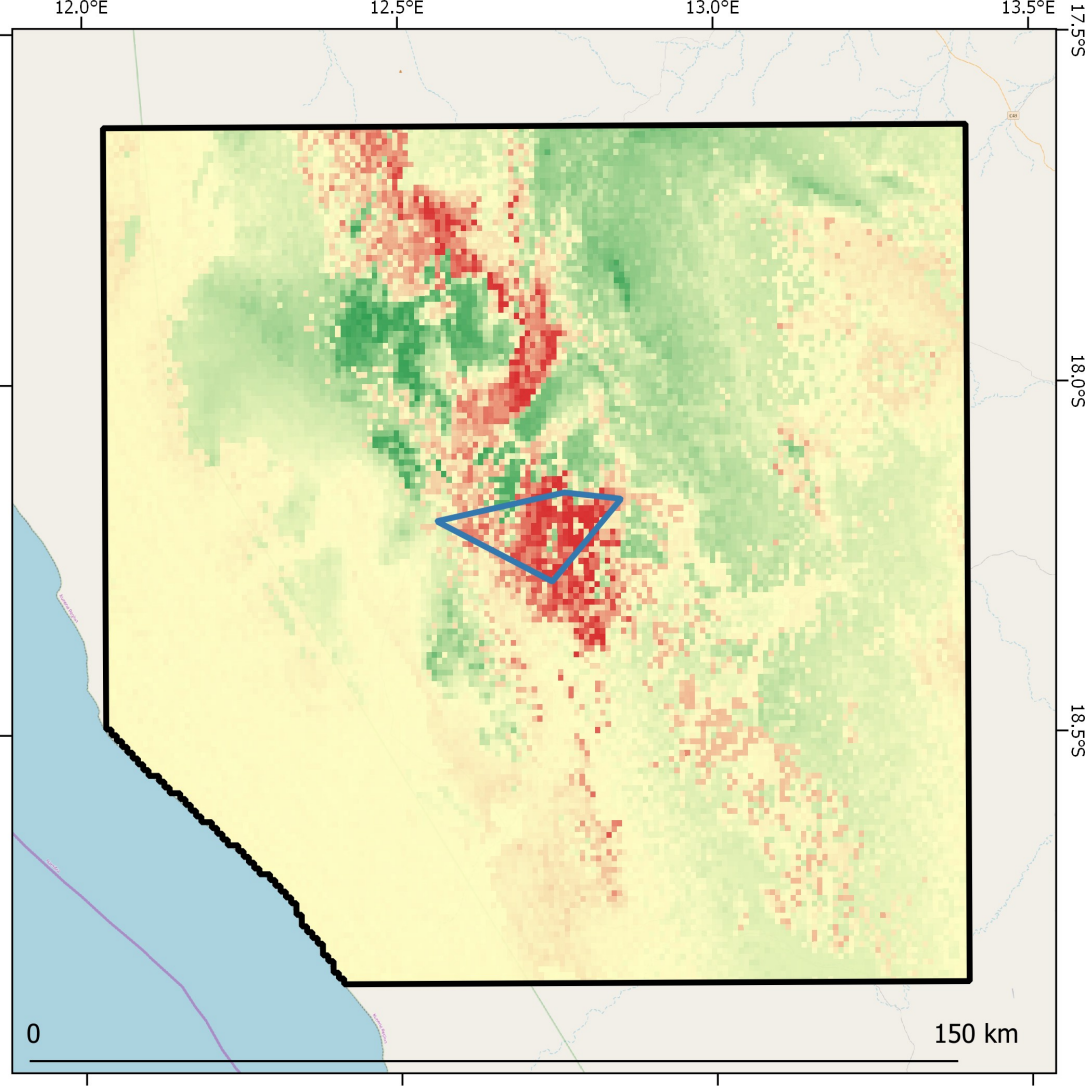
Variation of climatic suitability for *W*. *mirabilis* in the study area. Expected suitability variation from climate change (red and green shades indicate negative and positive variations respectively) calculated as the difference between future and historical suitability. Black polygon indicates the study area, and the blue polygon represents the boundaries of the observed extent of occurrence in Northern Namibia.

The strongest reduction of climatic suitability is expected in the eastern half of the extent of occurrence of welwitschia, as well as in some areas extending further north and south to the extent of occurrence (Fig 4). On the other hand, our models predict an increase in climatic suitability to the northwest of the current extent of occurrence (Fig 4). The MESS analysis indicated that all the pixels in the species extent of occurrence have positive values (minimum: 1.10, mean: 7.19 ± 2.47), suggesting that geographical extrapolation on future predictions are reliable (see S3 Fig). As a result, all the recorded stands are expected to be facing suitability reductions in the period 2000–2050, with variability between almost no reduction and complete reduction.

### Observed vs expected patterns

**Fig 5:**
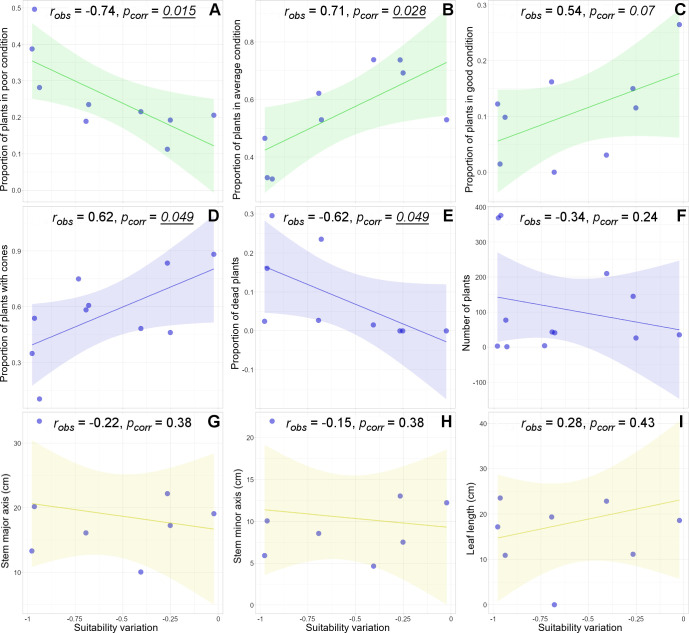
Linkage between population features and the expected suitability variation. Plots of the proportion of plants in poor (A), average (B), and good conditions (C), the proportion of plants with cones (D), the proportion of dead plants (E), the number of plants (F), the stem major (G) and minor axis (H), and the leaf length (I) as functions of the expected suitability variation in the stands. In all the plots, blue dots are values for plant stands, colored lines and areas represent the regression lines and the confidence intervals respectively, and the texts on the top of the plots indicate the results of the null-model correlation analyses (Pearson *r* and corrected *p* values for multiple comparisons). Note that suitability variation values (X-axis) are all negative; thus, the reduction of suitability increases from right to left.

Stronger predicted reductions of climatic suitability in the stand sites are associated with lower plant health condition, fewer plants with cones, and an increased number of dead plants. More specifically, the proportion of plants in poor condition in each stand increases with the reduction of suitability (Fig 5A). In contrast, the proportion of plants in average and good condition decreases as suitability variation decreases (Fig 5B and 5C). The proportion of plants with cones (i.e. a proxy of the potential population recruitment) is lower in stands where stronger reductions of climatic suitability are expected (Fig 5D). At the same time, the proportion of dead plants (i.e. population mortality) is negatively correlated with the predicted variation of climatic suitability (Fig 5E). However, neither the number of plants per stand (i.e. population size) (Fig 5F) nor plant body size (Fig 5G, 5H and 5I) is correlated with the suitability variation.

The observed geographic combination of species response does not follow any simple geographic gradient. Indeed, the latitude of welwitschia stands is not correlated with any measured variable (Table 1). Similarly, altitude and the measured variables are not correlated (Table 1). Overall, no latitudinal or altitudinal variation is occurring as a response to climate change.

**Table 1:** Linkage between population features and the stand latitude and altitude. Results of the null-model correlation analyses (Pearson *r* and corrected *p* values for multiple comparisons).

	Latitude		Altitude	
	*r*_*obs*_	*p*_*corr*_	*r*_*obs*_	*p*_*corr*_
Proportion of plants in poor conditions	-0.342	0.496	0.293	0.332
Proportion of plants in average conditions	-0.083	0.375	0.039	0.33
Proportion of plants in good conditions	0.258	0.425	-0.418	0.463
Proportion of plants with cones	0.425	0.151	-0.492	0.201
Proportion of dead plants	0.275	0.356	-0.048	0.066
Number of plants	-0.669	0.06	0.096	0.461
Stem length	0.521	0.26	-0.131	0.461
Stem width	0.542	0.177	-0.279	0.225
Leaf length	-0.243	0.102	0.573	0.3765

## Discussion

The observed pattern of population conditions of welwitschia plants in northern Namibia is consistent with the expected response under climate change, and specifically with predicted variations of climatic suitability. These results strongly suggest that ongoing climate change is affecting the status of welwitschia populations in the area and producing significant changes (i.e. contraction) of the species distribution at the local scale, which translates into a threat for the long-term conservation of the species. On the other hand, the geographic combination of welwitschia response to climate change does not follow a latitudinal or altitudinal gradient. Therefore, the potential impact of climate change on this species would have passed undetected using the common testing approach based on poleward/upwards range shift.

Inter-stand variations of multiple parameters (i.e. plant health conditions and proxies for population trends) are highly correlated with estimated variation in climatic suitability, strongly suggesting a negative effect of the ongoing climate change on welwitschia trees. In this light, the high correlation between the variation of climatic suitability and plant conditions (poor, average, and good) can support a link among climate change, the distribution of plants, and the variation of plant health with observed increase of individuals in poor conditions and the reduction of plants in average or good conditions. The loss of climatic suitability could also affect future population trends, by affecting recruitment (as suggested by the observed reduction of plants with cones) and mortality (as suggested by the observed increase of dead plants). Although we measured static parameters of population condition, the geographic combination of these parameters (observed in 2019) is coherent with the dynamism of a range shift from areas that were suitable in the past (before 2000) to areas that will be suitable in the future (2050). Indeed, bad population conditions such as poor plant health, low reproduction potential, and high mortality provide a proxy of negative population dynamics, which are typically associated with the trailing edge of species range. This study indicates a clear relationship between observed and expected patterns of populations response in welwitschia. The responsivity of welwitschia to climate change as measurable by proxies of population status could make this species a ‘sentinel’ of climate change effects for Namib ecosystems. The application of the approach used in this study is thus encouraged to identify sentinel species in other desert environments around the world for an effective biomonitoring of climate-linked biotic changes.

The visual estimation of plant condition can be considered a rough estimation of chlorophyll content of the leaves and thus of the plant’s photosynthetic efficiency [41,42]. Alterations of photosynthesis is a well-known effect of environmental stress [43,44]. Heat stress in particular inhibits photosynthesis in tropical and subtropical plants [68,69]. This effect can be stronger in arid environments, where water shortage can hamper the leaf temperature mitigation [70]. On the other hand, studies on other populations of *W*. *mirabilis* showed that rainfall is followed by an increase in the plant’s health status [40]. As a result, we hypothesize that the observed worsening of plant condition is associated with the complex interaction between the significant increase in temperature, which is the main predicted climate alteration occurring in the area (Fig 2), and the constant but limited water availability in the desert environment. However, specifically designed experiments would be needed to tease apart the different possible forces that could cause the observed responses.

Our results are consistent with the study of Bombi [23] carried out at the national level and at a much coarser spatial resolution. That study suggested a potential impact of climate change on welwitschia populations in northern Namibia and, predicted a general reduction of climatic suitability for *W*. *mirabilis* as well as potential effects on population recruitment and thus on population structure. The author postulated that adult welwitschia plants are likely to survive the expected reduction in climate suitability. In contrast, the correlation we observed between mortality and climate change would indicate a less optimistic scenario, with a progressive reduction of plant health, which translates in a potential long-term reduction of population size. This evidence could support the hypothesis that climate is changing faster and/or is becoming too hot/arid even for *W*. *mirabilis*. In this regard, it is worth noting that *W*. *mirabilis* is not a typical desert-adapted plant (it has C3 metabolism and a relatively high water demand [71]), and its ancestors probably occupied much more mesic habitats (probably even forests). It is likely that the current range fragmentation was the result of strong aridification during the Tertiary and Quaternary [72]; with current climate change predictions pointing towards further desertification (increase of 2°C in annual mean temperature is expected by 2050). Furthermore, this evidence should encourage specific management plans for northern Namibian populations and suggests that climate change should be considered among welwitschia conservation issues.

Quantitative data on plant physiological status (e.g. leaf growth rate, photosyntetic efficiency, water use efficiency), on substrate characteristics such as soil moisture profiles, and on population demographic parameters (e.g. annual recruitment, plant growth, annual mortality) are required to obtain a more detailed picture of the occurring alterations and to clarify the possible mechanistic linkage with climatic stress. Repeated physiological and demographic measurements in different sites would make it possible to follow plant responses over time. The activation of programs for the long-term monitoring of the species in the region would be particularly helpful, allowing critical situations to be detected at early stages and planning of effective recovery measures. Obviously, long-term monitoring in this remote area would be difficult and would require the involvement of local communities as well as the provision of significant resources by local and international agencies aimed at the conservation of desert ecosystems in Namibia.

Despite the great interest in *W*. *mirabilis*, which is the only living representative of an ancient lineage of gymnosperms [73], and its key-role in the Namib Desert ecosystems, several aspects of the species distribution and biology are still to be clarified for a science-based conservation strategy. First, the real level of geographic and genetic isolation of the different subranges should be verified in order to identify intra-specific evolutionary and conservation units. Second, an effort to census and make available the current knowledge on species distribution, demography, and conservation should be undertaken. Third, an analysis of climate change impacts should be extended to the other subranges and a science-based assessment of the conservation status should be made at local and global level. This set of measures could significantly contribute to conservation measures for the species that are effective in the long term.

The geographic combination of response we observed in welwitschia is more complex than the simple poleward/upward shift that was often observed for other species [33,35,74]. In the case of *W*. *mirabilis* populations of northern Namibia, the observed pattern of population conditions, which can represent a response to climate change, follows local contingencies rather than a simple latitudinal or altitudinal trends (Table 1). This could be associated with the small scale of the study, which emphasized the role of local factors (e.g. land morphology, dominant winds, recurring fog), but is also in agreement with previous large-scale studies. Previous studies pointed out that specific responses to climate change can be divergent [36] and that assuming a simplified poleward/upward species movement can result in climate change impacts being underestimated [37]. In our specific case, the linkage between climate change and population conditions, which is suggested by our results, would have been completely undetected with a simplified, but frequently used approach based on the assumption of poleward/upward shifts.

The comparison of the expected pattern of response to ongoing climate change, as estimated by suitability modeling, with the observed patterns of population conditions, as measured in the field, appeared as a powerful approach for detecting impacts of climate change on wild species. This approach, proposed by Bombi et al. [38], allowed the identification of climate change as potentially a major driver of the geographical pattern of welwitschia health, reproductive status, and size we observed in the field. Clearly, such an approach is prone to a certain level of false negative when other factors, not directly related to climate change (e.g. wind, herbivory, parasites), interact, blurring the pattern generated by climate change [38]. In addition, our small sample size could further increase the probability of type II errors. On the other hand, the probability of false positives is very low and not influenced by non-climatic factors [38] or sample size, strengthening the meaning of the detection of a climate change-related pattern. This study underlines the importance of considering species responses to climate change as an emergent property of the different effects on individual populations. At a higher biodiversity level, ecosystem responses to climate change can be considered as an emergent property of the effects on individual species. Such a hierarchical relationship provides direction for the application of spatially explicit approaches, such as the one used in this study, to multiple species and across diverse ecosystems. We therefore advocate for the implementation of a large-scale program for the identification of sentinel species of climate change effects. This would allow the detection, estimation, and monitoring of climate change impacts on biodiversity, improving the long-term conservation of species at the ecosystem level.

Our study confirms that desert-adapted species can be vulnerable to the effects of climate change. Although some studies have hypothesized minor impacts on desert ecosystems by climate change [75,76], growing evidence, provided through different approaches, shows severe effects of climate change on species, communities, and ecosystems in arid regions worldwide [18,77,78]. Altogether these studies, in agreement with our results, indicate that desert ecosystems are likely to suffer from biodiversity loss with intensifying global warming as result of a reduction of environmental suitability for the endemic biota [22].

## Supporting information

S1 Fig Examples of plants in the four health condition classes (A: Dead; B: Poor; C: Average; D: Good).
(PDF)

S2 Fig Variability of retained climatic parameters in the study area. (DOCX)

S3 Fig Map of multivariate environmental similarity surface. Color shades indicate the extent to which the predictor variables in each pixel are similar to the conditions experienced by the species in the presence sites. The black line separates zones with positive values to those with negative values. Negative MESS values indicate areas where at least one variable is outside the range of the experienced conditions and thus where model predictions can be less robust. The blue polygon represents the species extent of occurrence in the area.
(DOCX)

S1 Table Summary statistics of biotic variables for plants belonging to each health condition class. (DOCX)

S1 Appendix Biomod script. (DOCX)
