# Supplementary material for: Climate change effects on desert ecosystems: A case study on the keystone species of the Namib Desert Welwitschia mirabilis
Source: PLoS One. 2021 Nov 8;16(11):e0259767. doi: 10.1371/journal.pone.0259767 (PMC8575257; doi:10.1371/journal.pone.0259767)
Supplement: S1 Fig — Examples of plants in the four health condition classes (A: Dead; B: Poor; C: Average; D: Good). (PDF) [file pone.0259767.s001.pdf]

Climate change effects on desert ecosystems: a case study on the keystone species of the Namib Desert *Welwitschia mirabilis*

S1 Fig.

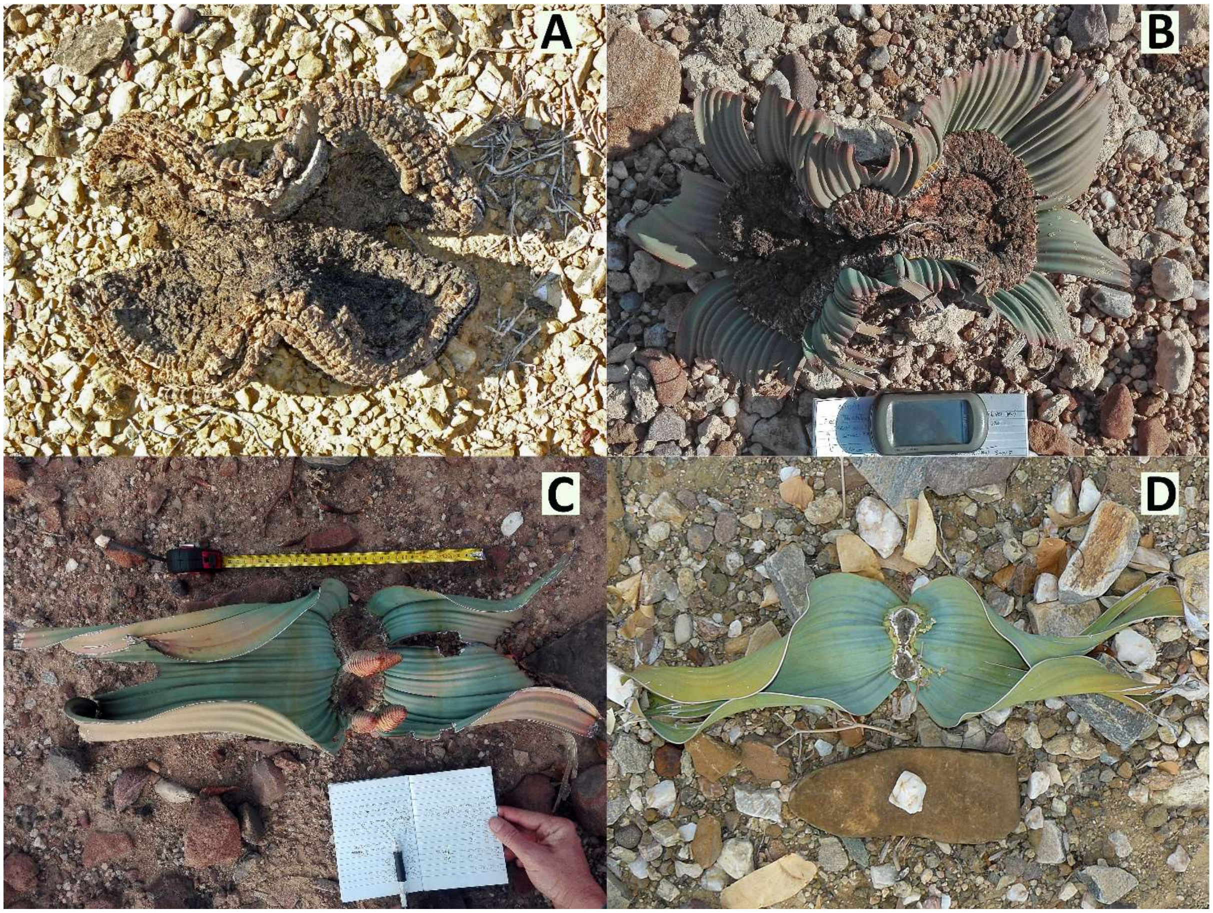

Examples of plants in the four health condition classes (A: dead; B: poor; C: average; D: good).
