# Supplementary material for: Climate change effects on desert ecosystems: A case study on the keystone species of the Namib Desert Welwitschia mirabilis
Source: PLoS One. 2021 Nov 8;16(11):e0259767. doi: 10.1371/journal.pone.0259767 (PMC8575257; doi:10.1371/journal.pone.0259767)
Supplement: S2 Fig — (DOCX) [file pone.0259767.s002.docx]

**Climate change effects on desert ecosystems: a case study on the keystone species of the Namib Desert *Welwitschia mirabilis***

S2 Fig.


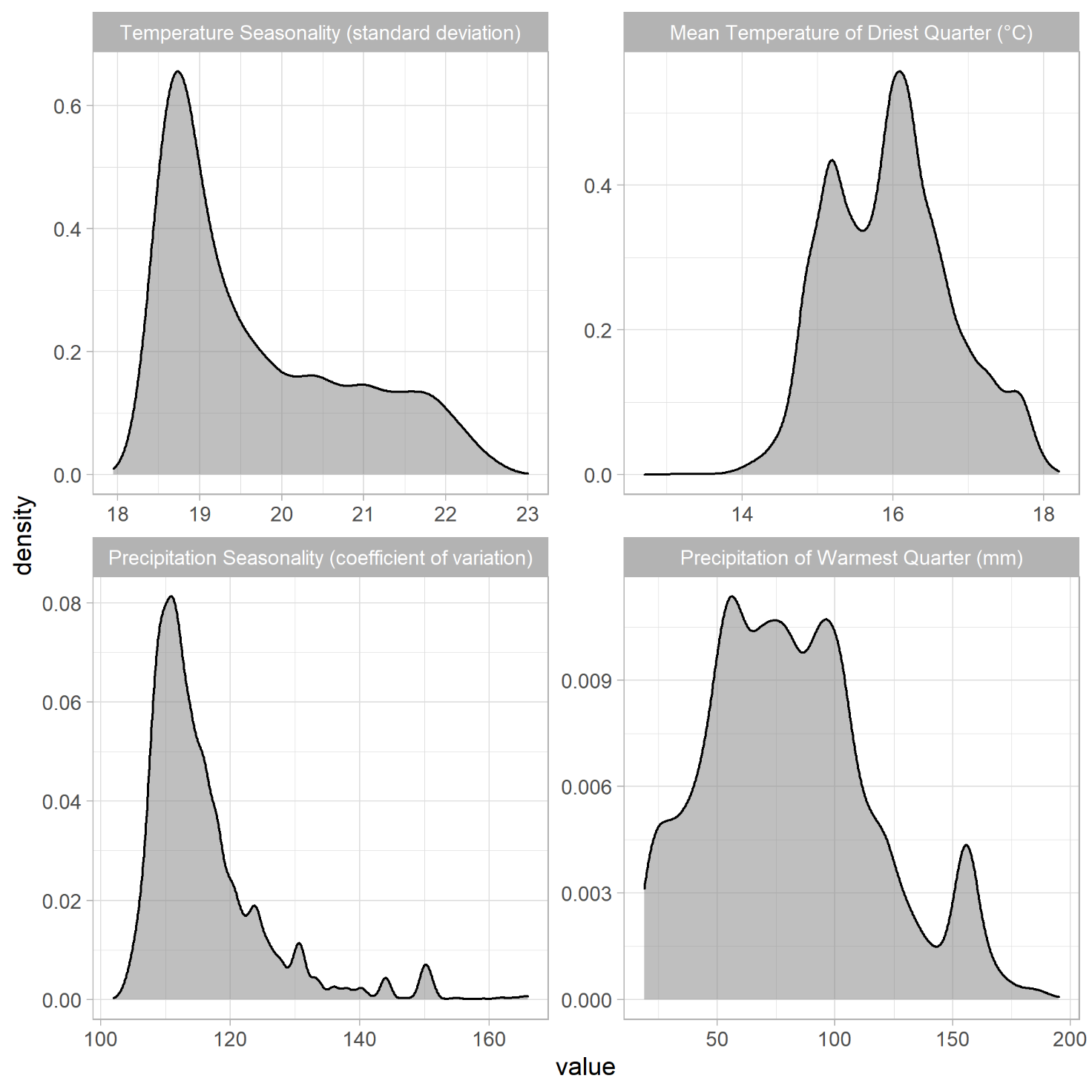


19.67 ± 1.14

15.97 ± 0.81

81.95 ± 35.61

115.94 ± 9.05

Variability of retained climatic parameters in the study area. Numbers are mean ± standard deviation.
