# Supplementary material for: Climate change effects on desert ecosystems: A case study on the keystone species of the Namib Desert Welwitschia mirabilis
Source: PLoS One. 2021 Nov 8;16(11):e0259767. doi: 10.1371/journal.pone.0259767 (PMC8575257; doi:10.1371/journal.pone.0259767)
Supplement: S3 Fig — Color shades indicate the extent to which the predictor variables in each pixel are similar to the conditions experienced by the species in the presence sites. The black line separates zones with positive values to those with negative values. Negative MESS values indicate areas where at least one variable is outside the range of the experienced conditions and thus where model predictions can be less robust. The blue polygon represents the species extent of occurrence in the area. (DOCX) [file pone.0259767.s003.docx]

**Climate change effects on desert ecosystems: a case study on the keystone species of the Namib Desert *Welwitschia mirabilis***

S4 Fig.


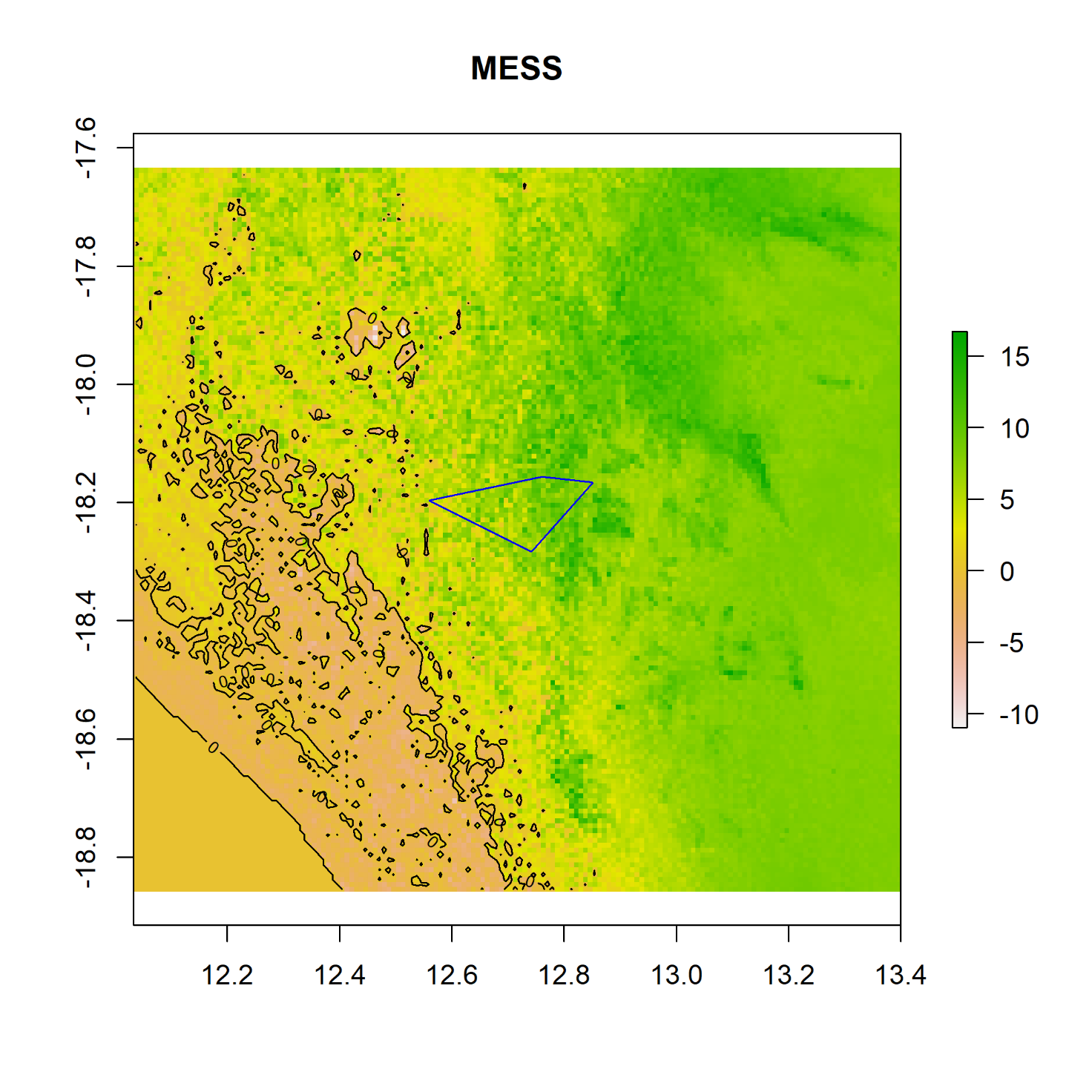


**Map of Multivariate Environmental Similarity Surface.** Color shades indicate the extent to which the predictor variables in each pixel are similar to the conditions experienced by the species in the presence sites. The black line separates zones with positive values to those with negative values. Negative MESS values indicate areas where at least one variable is outside the range of the experienced conditions and thus where model predictions can be less robust. The blue polygon represents the species extent of occurrence in the area.
