# Supplementary material for: Climate change effects on desert ecosystems: A case study on the keystone species of the Namib Desert Welwitschia mirabilis
Source: PLoS One. 2021 Nov 8;16(11):e0259767. doi: 10.1371/journal.pone.0259767 (PMC8575257; doi:10.1371/journal.pone.0259767)
Supplement: S1 Table — (DOCX) [file pone.0259767.s004.docx]

**Climate change effects on desert ecosystems: a case study on the keystone species of the Namib Desert *Welwitschia mirabilis***

S3 Tab.

| *Health condition* | *Stem major axis*  *(mean ± st. dev.)* | *Stem minor axis*  *(mean ± st. dev.)* | *Leaf length*  *(mean ± st. dev.)* | *Sex ratio* | *Prop. of plants with cones* |
| --- | --- | --- | --- | --- | --- |
| Dead | 27.65 ± 7.06 | 16.50 ± 6.55 |  |  |  |
| Poor | 17.65 ±14.22 | 8.98 ± 8.87 | 13.44 ± 9.84 | 1.08 | 0.315789 |
| Average | 18.04 ±13.82 | 9.80 ± 9.59 | 19.85 ± 13.10 | 1.18 | 1.017751 |
| Good | 22.03 ±15.52 | 12.75 ± 11.08 | 24.03 ± 17.24 | 1.60 | 1.653846 |

Summary statistics of biotic variables for plants belonging to each health condition class.
