# Supplementary material for: Climate change effects on desert ecosystems: A case study on the keystone species of the Namib Desert Welwitschia mirabilis
Source: PLoS One. 2021 Nov 8;16(11):e0259767. doi: 10.1371/journal.pone.0259767 (PMC8575257; doi:10.1371/journal.pone.0259767)
Supplement: S1 Appendix — (DOCX) [file pone.0259767.s005.docx]

**Climate change effects on desert ecosystems: a case study on the keystone species of the Namib Desert *Welwitschia mirabilis***

S5 Appendix

# model species distribution with biomod2 ==============================================

#generate random points

rp <- as.SpatialPoints.ppp(runifpoint(1000, win = study.area))

ext.occ.r <- rasterize(extent.occurrence, current.climate[[1]], field = rep(1, nrow(extent.occurrence)), background=0)

sp.presabs <- extract(ext.occ.r, rp)

# remove collinear variables

var.sel <- usdm::vifstep(current.climate, th=5)

# formatting data for biomod analyses

sp.clim <- extract(current.climate [[as.vector(var.sel@results[,1])]], rp)

Biomod.Data <- BIOMOD_FormatingData(resp.var=sp.presabs, expl.var=sp.clim,

resp.xy= coordinates(rp), resp.name="Welwitschia")

# Computing the models

Biomod.Model <- BIOMOD_Modeling(Biomod.Data, models=c('GLM','GBM','GAM','CTA','ANN','RF'),

models.options= BIOMOD_ModelingOptions(),

NbRunEval=2, DataSplit=80, Prevalence=0.5, VarImport=3,

models.eval.meth=c('TSS','ROC'))

# get all models evaluation

Biomod.Eval <- get_evaluations(Biomod.Model)

# Ensemble modeling

Biomod.EM <- BIOMOD_EnsembleModeling(modeling.output=Biomod.Model, chosen.models='all',

em.by='all', eval.metric=c('TSS'),

eval.metric.quality.threshold=c(0.7),

prob.mean.weight=T, prob.mean.weight.decay='proportional')

# projection over the current climate of the single models

pr.clim <- stack(current.climate[[as.vector(var.sel@results[,1])]])

Biomod.Proj <- BIOMOD_Projection(modeling.output=Biomod.Model, new.env=pr.clim,

proj.name='current', selected.models='all')

# projection over the current climate of the ensemble models

Biomod.EF <- BIOMOD_EnsembleForecasting(EM.output=Biomod.EM, projection.output=Biomod.Proj)

# projection over the future climate

for(i in 1:59){ # 59 scenarios for future climate were produced under CMIP5

sel.scen <- available.scenarios[[i]]

new.clim = sel.scen[[as.vector(var.sel@results[,1])]]

FT.Biomod.Proj <- BIOMOD_Projection(modeling.output=Biomod.Model, new.env=new.clim)

FT.Biomod.EF <- BIOMOD_EnsembleForecasting(EM.output=Biomod.EM,

projection.output=FT.Biomod.Proj)

}
